# Development and Validation of a HPLC and an UV Spectrophotometric Methods for Determination of Dexibuprofen in Pharmaceutical Preparations

**DOI:** 10.5402/2011/948314

**Published:** 2011-07-05

**Authors:** Selvadurai Muralidharan, Subramania Nainar Meyyanathan

**Affiliations:** Department of Pharmaceutical Analysis, JSS College of Pharmacy, Ootacamund, Tamilnadu-643 001, India

## Abstract

A high-performance liquid chromatographic (HPLC) and a ultraviolet (UV) methods were developed and validated for the quantitative determination of Dexibuprofen (DI) in pharmaceutical dosage form. HPLC was carried out by reversed phase technique on a RP-18 column with a mobile phase composed of acetonitrile and 0.5% triethylamine (pH 7.5 adjusted with orthophosphoric acid (30 : 70, v/v)). UV method was performed with the *λ* max at 222.0 nm. Both the methods showed good linearity, reproducibility and precision. No spectral or chromatographic interferences from the tablet excipients were found in UV and HPLC. The method was successfully applied to commercial DEXIFEN tablets. Validation parameters such as linearity, precision, accuracy, and specificity were determined. The proposed method could be applicable for routine analysis of DI and monitoring of the quality of marketed drugs.

## 1. Introduction

Nonsteroidal anti-inflammatory drugs (NSAIDs) like ± ibuprofen are effective for the relief of pain, and their use is widespread [1–3]. ± ibuprofen, which contains equal quantities of (+) Ibuprofen and (−) Ibuprofen, has been used as an anti-inflammatory and analgesic agent for over 30 years. (+) Ibuprofen, or Dexibuprofen, is the pharmacologically effective enantiomer of + ibuprofen [1–3]. The present UV and HPLC methods are relatively simple, rapid, and highly sensitive in the determination of dexibuprofen. 

Only limited methods have been reported in the literature survey [4–10]. The aim of the present work was to develop and validate a simple, fast, and reliable isocratic RP-HPLC and UV method for the determination of DI in pharmaceutical dosage forms. The important features and novelty of the proposed method included simple sample treatment with sonication of small amount of powder sample at ambient temperature, centrifugation, and dilution; short elution time with internal standard eluted prior to DI; good precision (R.S.D. less than 5%) and high recovery (greater than 95%). Confirmation of the applicability of the developed method was validated according to the International Conference on Harmonisation (ICH), to determination of DI in pharmaceutical dosage forms.

## 2. Experimental

### 2.1. Chemicals

HPLC grade acetonitrile (ACN) and triethylamine (A.R. grade) were purchased from Qualigens Fine Chemicals, Mumbai. Water HPLC grade was obtained from a Milli-QRO water purification system. Dexibuprofen standard and ibuprofen (Internal standard) were provided by Noven Life Sciences Private Limited, Hyderabad, India.

### 2.2. Instrumentation and Analytical Conditions

HPLC chromatographic separation was performed on a Shimadzu liquid chromatographic system equipped with a LC-10AT-vp solvent delivery system (pump), SPD M-10AVP photo diode array detector, and Rheodyne 7725i injector with 50 *μ*L loop volume. Class-VP 6.01 data station was applied for data collecting and processing (Shimadzu, Japan). The HPLC was carried out at a flow rate of 1.0 mL min^−1^ using a mobile that is phase constituted of acetonitrile—50 mM potassium dihydrogen orthophosphate (pH 7.5 adjusted with orthophosphoric acid (45 : 55, v/v)), and detection was made at 222.0 nm. The mobile phase was prepared daily, filtered through a 0.45 *μ*m membrane filter (Millipore) and sonicated before use. A Princeton SPHER C_18_ column (25 cm × 4.6 mm i.d., 5 *μ*) was used for the separation. UV method was performed on a UV-VISIBLE spectrophotometer, UV-160 (Shimadzu) with the *λ* at 222.0 nm and using 1.0 cm quartz cell.

### 2.3. Preparation of Standard Solutions

#### 2.3.1. HPLC Method

For the calibration curve, accurately weighed 100.0 mg of DI was transferred to a 100 mL volumetric flask and dissolved in a mixture of water and methanol of the ratio 1 : 1 v/v. From this solution, other solutions with concentrations of 10.0, 20.0, 30.0, 40.0, 50.0, and 60.0 *μ*g mL^−1^ were obtained by diluting adequate amounts in triplicate.

#### 2.3.2. UV Method

For the calibration curve, accurately weighed 100.0 mg of DI was transferred to a 100 mL volumetric flask and dissolved in a mixture of water and methanol of the ratio 1 : 1 v/v. From this solution, other solutions with concentrations of 2.0, 4.0, 6.0, 8.0, 10.0, and 12.0 *μ*g mL^−1^ were obtained by diluting adequate amounts in triplicate.

### 2.4. Preparation of Sample Solutions

#### 2.4.1. HPLC Method

Twenty tablets, each containing 200.0, 300.0, and 400.0 mg of DI were weighed and finely powdered; a quantity of powder equivalent to 20.0, 30.0 and 40.0 mg of DI was weighed and transferred to a sintered glass crucible. To this 5.0 mL of 1.0 mg mL^−1^ solution of ibuprofen was added and the drugs were extracted with three quantities, each of 20 mL of mixture of methanol and water (1 : 1 v/v). The combined extracts were made up to 100 mL with mobile phase, and further dilutions were made to get a concentration of 20.0, 30.0, and 40.0 *μ*g/mL of dexibuprofen, 50.0 *μ*g/mL of Ibuprofen as internal standard, and this solution was used for the estimation.

#### 2.4.2. UV Method

Accurately weighed amount of powder equivalent to 20.0, 30.0, and 40.0 mg of DI was transferred to 100 mL volumetric flask and dissolved in the mobile phase to obtain a concentration of 20.0, 30.0, and 40.0 *μ*g mL^−1^. An aliquot of this solution was diluted in mobile phase to obtain a solution with final concentration of 4.0 *μ*g mL^−1^.

### 2.5. Method Validation

The objective of method validation is to demonstrate that the method is suitable for its intended purpose as it is stated in ICH guidelines [11]. The method was validated for linearity, precision (repeatability and intermediate precision), accuracy specificity, short-term stability, and system suitability. Standard plots were constructed with six concentrations in the range of 10–60 *μ*g mL^−1^ prepared in triplicates to test linearity. The ratio of peak area signal of DI to that of IS was plotted against the corresponding concentration to obtain the calibration graph. The linearity was evaluated by linear regression analysis that was calculated by the least square regression method. The precision of the assay was studied with respect to both repeatability and intermediate precision. Repeatability was calculated from six replicate injections of freshly prepared DI solution in the same equipment at a concentration 50 *μ*g mL^−1^ of the intended test concentration value on the same day. The experiment was repeated by assaying freshly prepared solution at the same concentration additionally on two consecutive days to determine intermediate precision. Peak area ratio of DI to that of IS was determined and precision was reported as % R.S.D. Method accuracy was tested (% recovery and % R.S.D. of individual measurements) by analysing samples of DI at three different levels in pure solutions using three preparations for each level. The results were expressed as the percentage of DI recovered in the samples. Specificity was assessed by comparing the chromatograms obtained from sample of pharmaceutical preparation and standard solution with those obtained from excipients which take part in the commercial tablets and verifying the absence of interferences. Sample solution short-term stability was tested at ambient temperature (20 ± 1°C) for three days. In order to confirm the stability of both standard solutions at 100% level and tablets sample solutions, both solutions protected from light were reinjected after 24 and 48 h at ambient temperature and compared with freshly prepared solutions. A system suitability test was performed by six replicate injections of the standard solution at a concentration of 50 *μ*g mL^−1^ verifying IS/DI resolution >2, % R.S.D. of peak area ratios of DI to that of IS ±2%, % R.S.D. of each peak retention time ±2%.

## 3. Results and Discussion

### 3.1. Validation of Methods

#### 3.1.1. Linearity

Six point calibration graphs were constructed covering a concentration range of 10–60 mg mL^−1^. Three independent determinations were performed at each concentration. Linear relationships between the ratio of the peak area signal of DI to that of IS versus the corresponding drug concentration were observed, as shown by the results presented in Table 1. The standard deviations of the slope and intercept were low. The determination coefficient (*r* ^2^) exceeded 0.99 (Figure 2).

#### 3.1.2. Precision

The carried out repeatability study (*n* = 6) showed a R.S.D. of 0.858% for the peak area ratios of DI to that of IS obtained, thus showing that the equipment used for the study worked correctly for the developed analytical method and is highly repetitive. For the intermediate precision, a study carried out by the same analyst working on two consecutive days (*n* = 3) indicated a R.S.D. of 0.744%. Both values were far below 5%, the limit percentage set for the precision, and indicated a good method precision.

#### 3.1.3. Accuracy

The data for accuracy were expressed in terms of percentage recoveries of DI in the real samples. These results are summarized in Table 2. The mean recovery data of DI in real sample were within the range of 100.01 and 102.28%, and mean % R.S.D. was 1.04%, satisfying the acceptance criteria for the study.

#### 3.1.4. Specificity

**Figure 1:**
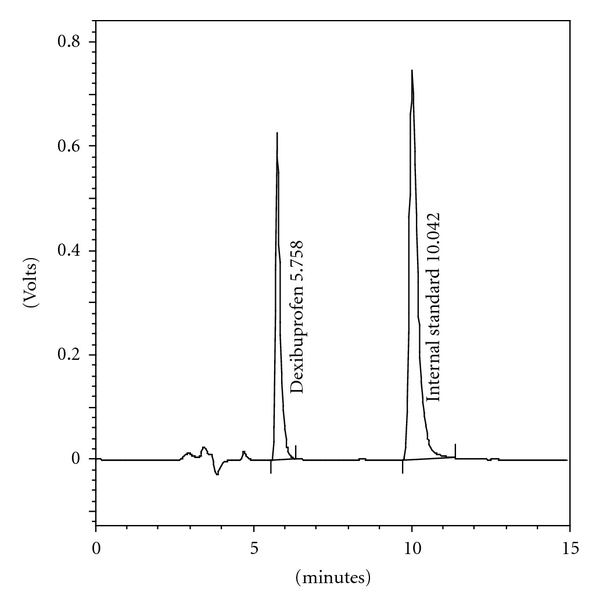
Typical sample chromatogram of dexibuprofen and internal standard.

**Figure 2:**
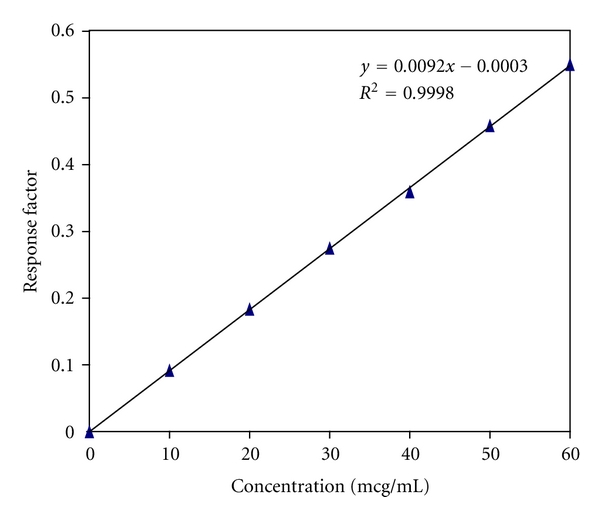
Calibration curve of dexibuprofen.

The HPLC chromatogram recorded for the mixture of the drug excipients revealed no peak within a retention time range of 5 min. The results showed that the developed method was specific as none of the excipients interfered with the analytes of interest (Figure 1).

#### 3.1.5. Stability

The stability of DI in standard and sample solutions containing IS was determined by storing the solutions at ambient temperature (20 ± 1°C) protected from light. The solutions were checked in triplicate after three successive days of storage, and the data were compared with freshly prepared samples. In each case, it could be noticed that solutions were stable for 72 h, as during this time the results did not decrease below 97%. This denotes that DI is stable in standard and sample solutions for at least 3 days at ambient temperature, protected from light, and is compatible with IS.

#### 3.1.6. System Suitability

The resolution factor between IS and DI, in the developed method, was above 2. The % R.S.D. of peak area ratio of DI to that of IS and retention times for both drug and IS were within 2% indicating the suitability of the system (Table 3). These results indicate the applicability of this method to routine with no problems, with its suitability being proved. The statistical evaluation of the proposed method revealed its good linearity, reproducibility, and its validation for different parameters and led us to the conclusion that it could be used for the rapid and reliable determination of DI in pharmaceutical forms.

#### 3.1.7. Assay of Tablets

The validated method was applied for the assay of commercial tablets containing 200 mg, 300 mg, and 400 mg of DI (DEXIFEN); each sample was analysed in triplicate after extracting the drug as mentioned in the assay sample preparation of Section 2 and injections were carried out in triplicate. Figure 1 shows an HPLC chromatogram of DI in pharmaceutical tablets. None of the tablets ingredients interfered with the analyte peak. The results presented in Table 4 are in good agreement with the labelled content.

## 4. Conclusion

A validated isocratic HPLC and UV methods has been developed for the determination of DI in dosage forms. The proposed method is simple, rapid, accurate, precise, and specific. Its chromatographic run time of 10.5 min allows the analysis of a large number of samples in a short period of time. Therefore, it is suitable for the routine analysis of DI in pharmaceutical dosage forms. The simplicity of the method allows for application in laboratories that lack sophisticated analytical instruments such as LC-MS/MS or GC-MS/MS that are complicated, costly, and time consuming rather than a simple HPLC-UV method. Considering the possible worldwide development of counterfeit DEXIFEN, the proposed method could be useful for the national quality control laboratories in developing countries.

## Figures and Tables

**Table 1:** Results of regression analysis of data for the quantitative determination of dexibuprofen by the proposed methods.

Statistical parameters	HPLC	UV
Concentration range (*μ*g mL^−1^)	10–60	2–12
Regression equation	*y* = 0.0186*x* + 0.044	*y* = 0.1117 + 0.0095
Correlation coefficient (*r*)	0.9915	0.9973

**Table 2:** Accuracy study for Dexibuprofen (*n* = 5).

Nominal concentration *μ*g mL^−1^ (HPLC)	Nominal concentration *μ*g mL^−1^ (UV)	Mean recovery (%)		R.S.D. (%)	
		HPLC	UV	HPLC	UV
10.00	2.0	8.30	1.91	0.32	0.12
30.00	6.0	28.24	6.05	0.78	0.35
60.00	12.0	57.99	11.68	1.02	0.62

**Table 3:** System suitability study (HPLC).

Retention time (min)	DI (50 *μ*g/mL)	IS (10 *μ*g/mL)
Mean (*n* = 5)	7.53	11.12
% R.S.D.	0.39	0.13

**Table 4:** Results obtained for determination of dexibuprofen in SIBET marketed formulations.

	HPLC		UV		HPLC	UV
Drug	Amount mg/tab		Amount mg/tab		% Recovery	
	Labelled	Found	Labelled	Found		
	400	385.23	400	378.89	96.30	95.89
Dexibuprofen	300	291.98	300	285.67	97.32	93.12
	200	189.83	200	185.42	94.91	92.05
